# Enhancing functional properties and analysis of sugar and metabolite composition of *Hylocereus megalanthus* juice through *Bifidobacterium* fermentation

**DOI:** 10.1016/j.fochx.2025.102945

**Published:** 2025-08-21

**Authors:** Patthanan Sakda, Huixian Wang, Juanni Li, Jiangyue Zheng, Yuke He, Wanwipa Vongsangnak, Ruimin Wang

**Affiliations:** a School of Food Science and Engineering, Key Laboratory of Food Nutrition and Functional Food of Hainan Province, Hainan University, Haikou 570228, China; b Collaborative Innovation Center of One Health, Hainan University, Haikou 570228, China; c Interdisciplinary Graduate Program in Bioscience, Faculty of Science, Kasetsart University, Bangkok 10900, Thailand; d Department of Zoology, Faculty of Science, Kasetsart University, Bangkok 10900, Thailand

**Keywords:** *Hylocereus megalanthus*, *Bifidobacterium* fermentation, Metabolic profiles, Antioxidant activity, Sensory properties

## Abstract

*Bifidobacterium* fermentation has been shown to provide significant health benefits and safety advantages. This study investigated the impact of *Bifidobacterium* fermentation on the physicochemical characteristics, metabolic profiles, and sensory properties of *Hylocereus megalanthus* juice (HMJ). *Bifidobacterium* fermentation enhanced the physicochemical profile of HMJ by improving bioactive components and increasing the antioxidant activity. Notably, the increased soluble dietary fiber (SDF) and sodium cholate adsorption capacity (SCAC) of fermented HMJ possessed potential hypolipidemic effects. A total of 638 differential metabolites were identified by non-targeted metabolomics, mainly enriched in amino acid and carbohydrate metabolism. Targeted analysis of sugar metabolism further highlighted the transformation of mono-, di-, and tri-saccharides, particularly within the pentose phosphate pathway. Additionally, electronic tongue analysis and sensory evaluation demonstrated an improvement in the flavor profile of HMJ. These findings highlight the application of *Bifidobacterium* fermentation in enhancing the nutritional and sensory properties of HMJ, making it a more functional beverage.

## Highlights

• *Hylocereus megalanthus* juice (HMJ) serves as an effective fermentation substrate.
• *Bifidobacterium* fermentation enhanced physicochemical properties of HMJ.
• Fermentation boosted bioactive substances and bioactivities of HMJ.
• Fermentation altered metabolic profile and transformed sugar composition of HMJ.
• Electronic tongue test showed notable flavor improvement of fermented HMJ.

## 1 Introduction

*Hylocereus megalanthus* (yellow pitahaya), also known as yellow dragon fruit, is a member of the *Hylocereus* genus within the cactus family (Cactaceae). *Hylocereus megalanthus* is native to Americas and is now cultivated across the globe, particularly in tropical and subtropical regions (Valero et al., 2025). Since *Hylocereus megalanthus* introduction in Hainan province of China, the fruit has steadily gained recognition for its nutritional value and potential health benefits, attracting growing interest among consumers (Zhu et al., 2022). *Hylocereus megalanthus* is prized not only for its striking appearance and appealing flavor but also for its exceptional nutritional content. It contains high levels of soluble sugars, dietary fiber, proteins, antioxidants and bioactive compounds (Valero et al., 2025), as well as essential minerals such as potassium, magnesium, and calcium, which contribute to its rising popularity as a functional food (Viñas & Jiménez, 2016). However, while its nutritional benefits are well recognized, research on its applications in areas such as probiotic fermentation remains limited.

*Bifidobacterium* is a key genus in the human gut microbiota, highly susceptible to antibiotics (Reyman et al., 2022), and widely recognized as an important strain of lactic acid bacteria (LAB), the most well-known probiotics. It helps regulate gut microbiota, enhance immunity, improve metabolism, lower cholesterol, and promote anti-aging (Wang, He, et al., 2024). Fermentation with LAB is an emerging approach to enhance the nutritional and functional properties of fruits. LAB fermentation can improve the bioavailability of nutrients, increase the production of bioactive compounds, and enhance gut health benefits (Chen et al., 2023; Deba-Rementeria et al., 2023). Furthermore, LAB fermentation is known to degrade complex carbohydrates, making the fruit's sugars more digestible and potentially beneficial for individuals with metabolic disorders. Recent studies on fruit fermentation suggest that LAB fermentation can also increase polyphenol content, boost antioxidant activity and further enhance the fruit's potential as a functional food (Duan et al., 2023; Wang et al., 2021; Wang, Zhou, et al., 2024). A previous study revealed the mechanism of *Bifidobacterium animalis* fermentation on the metabolic activities and biotransformation of *Elaeagnus moorcroftii Wall. ex Schlecht* (Wang, Wang, et al., 2024).

Uncovering new bioactive metabolites by fermentation could broaden the fruit's applications in functional foods and nutraceuticals, aligning with the growing global demand for healthier dietary options. Additionally, research into the potential of *Hylocereus megalanthus* as a functional food, particularly in Hainan province, plays a crucial role in advancing the region's agricultural sector. This study investigated the functional properties, metabolites, and sensory changes of the *Hylocereus megalanthus* juice (HMJ) fermented by three *Bifidobacterium animalis* subsp. *lactis* (*B. lactis*) (H22B656, HNU329, and HNU212). The findings will contribute to a deeper scientific understanding of *Hylocereus megalanthus* and support its expansion as a versatile agricultural product with significant functional food applications.

## 2 Material and method

### 2.1 Materials and chemicals

*Hylocereus megalanthus* fruits were picked up from Lingshui, Hainan, China. After being thoroughly washed, the fruit flesh was manually crushed and squeezed to extract juice, which was then used as the fermentation matrix for subsequent analysis.

Formic acid and acetonitrile (LC-MS grade) were purchased from Merck (Darmstadt, Germany) and Thermo Fisher Scientific (USA), respectively. Sugar standards were provided by CNW, IsoReag, and TCI (Shanghai, China). Sugar standards solutions (2 mg/mL) were prepared in methanol (MeOH), stored at −20 °C, and diluted as needed before analysis.

### 2.2 Preparation of fermented HMJ

Three strains of *B. lactis* (H22B656, HNU329, HNU212) were isolated from healthy infant feces and employed as fermentation strains for HMJ. These strains were successively passaged to the third generation using a 2 % (*w*/w) inoculum, ensuring stability before use. The HMJ was pasteurized at 85 °C for 15 min. After cooling, each strain was inoculated into the sterilized HMJ at 4 % (*w*/w) inoculum and incubated under an anaerobic condition at 37 °C for 36 h to reach the fermentation endpoint. A batch of unfermented HMJ was maintained as a control for further comparison.

### 2.3 Physicochemical properties in HMJ

This study examined essential parameters affecting *Bifidobacterium* fermented HMJ, including pH, reducing sugars, titratable acidity (TA), soluble solids, and organic acids. The pH was measured with a calibrated digital pH meter to ensure precision. Titratable acidity (TA) was assessed through titration with 0.1 N sodium hydroxide and expressed as a percentage of citric acid equivalents (%). The concentration of reducing sugars was quantified via the dinitrosalicylic acid (DNS) method. The total soluble solids content was measured using a digital refractometer and expressed in degrees Brix (°Brix). Organic acid compounds were analyzed by high-performance liquid chromatography (HPLC) following previously method (Wang, He, et al., 2024).

### 2.4 Determination of bioactive substances contents in HMJ

The total phenolic content (TPC) was measured using the Folin-Ciocalteu method and expressed as gallic acid equivalents (GAE), while the total flavonoid content (TFC) was quantified via a colorimetric assay with aluminum chloride and expressed as rutin equivalents (RE) following our previous research (Wang, Wang, et al., 2022).

The HMJ samples were mixed with ultrapure water at a material-liquid ratio of 1:25 (g/mL), and the pH was adjusted to 4.8 ± 0.1. Cellulase was then added at a mass ratio of 1:1 (g/g, sample/cellulase) and incubated at 50 ± 1 °C for 4 h. Subsequently, the supernatant was centrifuged at 3500 r/min for 10 min to facilitate separation. Subsequently, 4 times the volume of 95 % food-grade ethanol was added, and the mixture was left overnight. The solution was then centrifuged again at 3500 r/min for 15 min. The s precipitate was collected, dried at low temperature, and weighed. The dried precipitate represented the soluble dietary fiber (SDF) extracted from the sample. The SDF content was calculated using the following equation:

SDF content (%) = SDF mass (g)/sample mass (g) × 100

### 2.5 Determination of bioactivities in HMJ

#### 2.5.1 Antioxidant activity

The antioxidant activity of HMJ was analyzed following established protocols (Wang, Xue, et al., 2024) with minor modifications. The assessment utilized three widely recognized assays, including 2,2-diphenyl-1-picrylhydrazyl (DPPH) free radical scavenging capacity, cupric-reducing antioxidant capacity (CUPRAC), and ferric-reducing antioxidant power (FRAP). Results were expressed as Trolox equivalents (TE).

#### 2.5.2 Measurement of sodium cholate adsorption capacity (SCAC) in HMJ

The cholate adsorption capacity was determined by the furfural colorimetric method (Zhang, Zhang, Guo, Ye, et al., 2023). Briefly, 0.2 g of sodium cholate was dissolved in a 15 mmol/L NaCl solution (pH = 7.0), followed by the addition of 1.0 g of lyophilized fermented fruit juice. The mixture was incubated at 37 °C with continuous shaking for 2 h to facilitate adsorption. Following incubation, the samples were subjected to centrifugation at 4000 ×*g* for 15 min, and the resulting supernatant (1 mL) was aliquoted for downstream analyses. This supernatant was mixed with 6 mL of 45 % sulfuric acid solution, followed by the addition of 1 mL of 0.3 % furfural solution. The reaction mixture was heated at 65 °C for 30 min, cooled to room temperature, and absorbance was measured at 620 nm. The standard curve was generated using sodium cholate as a reference, allowing for the precise quantification of cholate adsorption. The SCAC was calculated as follows:

SCAC (mg/g) = (m_1_-m_2_)/m.

where m_1_ is the cholate mass (mg) of the solution before adsorption; m_2_ is the mass (mg) of sodium cholate in the supernatant after adsorption; m is the mass (g) of the sample.

### 2.6 Non-targeted metabolomics analysis in HMJ

The metabolite profile of HMJ was analyzed using ultra high-performance liquid chromatography (UHPLC) coupled with a Q-Exactive high-resolution mass spectrometer (Thermo Fisher Scientific, California, USA). The differential metabolites was conducted based on the criteria of log2(Fold Change) ≥ 1 and *p*-value <0.05 between comparison groups. VIP (Variable Importance in Projection) >1 from the OPLS-DA (Orthogonal Partial Least Squares Discriminant Analysis) model was used to assess the contribution of each metabolite to the different groups. Further functional insights were obtained through the Kyoto Encyclopedia of Genes and Genomes (KEGG) and Human Metabolome Database (HMDB) analyses, providing a comprehensive understanding of their roles in metabolic pathways.

### 2.7 Targeted metabolomics analysis in HMJ

A 50 μL sample was mixed with 500 μL of methanol: isopropanol: water (3:3:2), vortexed for 3 min, ultrasonicated for 30 min, and centrifuged at 12,000 r/min at 4 °C for 3 min. The 50 μL supernatant was combined with 20 μL of an internal standard, evaporated under nitrogen gas, freeze-dried, and derivatized. Derivatization was conducted by incubating the sample with methoxyamine hydrochloride in pyridine at 37 °C for 2 h, followed by the addition of BSTFA and further incubation at 37 °C for 30 min. The final mixture was analyzed via Gas chromatography-mass spectrometry (GC–MS) analysis, which was performed using an Agilent 7890B GC system coupled with a 7000D mass spectrometer and a DB-5MS column. Helium was employed as the carrier gas at a flow rate of 1 mL/min, with an injection volume of 2 μL with a split ratio of 3:1. Metabolite selection was based on absolute Log2 fold change (Log_2_FC). KEGG annotation and pathway enrichment analysis were conducted by mapping identified metabolites to metabolic pathways, followed by Metabolite Set Enrichment Analysis (MSEA) using hypergeometric tests to determine the enriched metabolic functions.

### 2.8 Sensory profile analysis

Sensory profiling of HMJ was performed using an electronic tongue (E-tongue) system (SA402B, Insent, Japan), which was featured a multichannel sensor array capable of detecting and quantifying bitterness, astringency, sourness, aftertaste-astringency (aftertaste-A), aftertaste-bitterness (aftertaste-B), richness, umami, and saltiness. Data acquisition was conducted in real time using an integrated logging platform, ensuring accurate and reliable sensory measurement. In addition, sensory evaluation was performed using a 9-point hedonic scale. Specifically, sensory analysis was conducted with 14 trained panelists (7 males and 7 females, aged 20-40 years), who evaluated both fermented and non-fermented HMJ for seven key sensory attributes (color, odor, sweetness, sourness, astringency, fermented flavor, and overall acceptability). Scores were averaged across panelists and presented as radar chart for comparative visualization.

### 2.9 Statistical analysis

Each experimental condition was independently replicated three times, and quantitative data are presented as mean ± standard deviation (SD). Multivariate statistical analysis, hierarchical clustering (heat map), chord diagram visualization, and correlation analysis were performed using R (version 4.2.2). Statistical calculations and hypothesis testing were performed using SPSS 25.0 (SPSS, Inc., USA), while Principal component analysis (PCA) was executed using OriginPro 2018 (Origin, Inc., USA). Bar plots and pie charts were generated using GraphPad Prism 10 (GraphPad, Inc., USA). To determine statistical significance, one-way analysis of variance (ANOVA) followed by Duncan's test was applied, with *p* < 0.05 considered statistically significant.

## 3 Results and discussion

### 3.1 Physicochemical properties of fermented HMJ

**Fig. 1:**
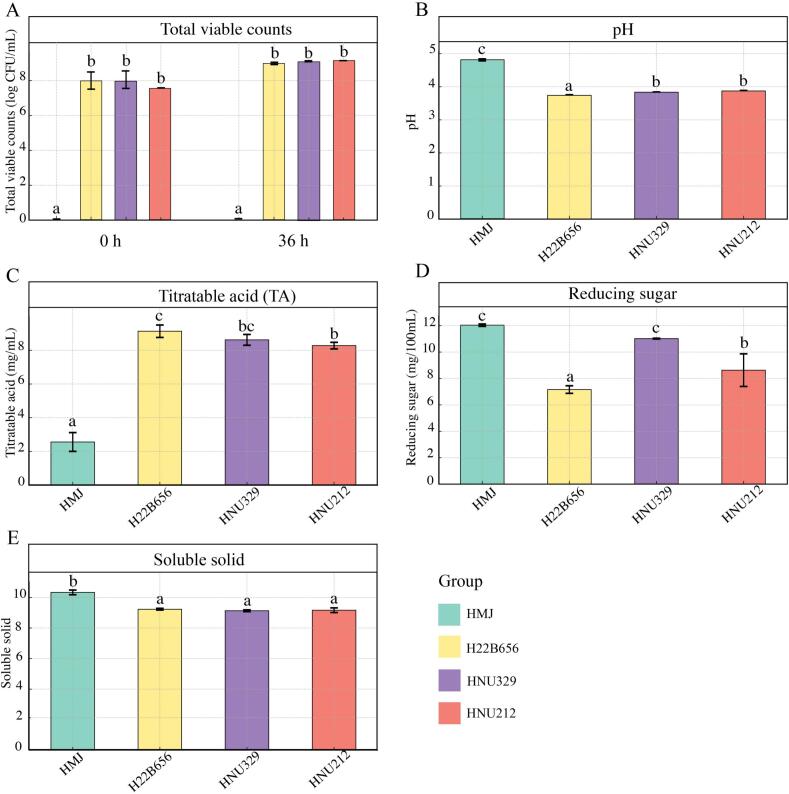
Change in physicochemical properties. Total viable counts (A); pH (B); titratable acid (TA) (C); reducing sugar (D); and soluble solid (E) of HMJ and LAB fermented HMJ. Different lowercase letters indicate significant differences at the *p* < 0.05 level.

The physicochemical properties of HMJ are presented in Fig. 1. The total viable count is recognized as one of the most reliable indicators of substrate adaptability in LAB strains (Chen et al., 2023). Following fermentation, the total viable count of fermented HMJ showed a significant increase (*p* < 0.05), as indicated in Fig. 1A. After 36 h of fermentation, HMJ fermented with HNU212 showed the highest total viable count (9.16 ± 0.03 log CFU/mL), followed by HMJ fermented with HNU329 (9.09 ± 0.05 log CFU/mL), and HMJ fermented with H22B656 (8.99 ± 0.08 log CFU/mL), respectively. However, no significant differences of total viable count were observed between HMJ-fermented groups after 36 h fermentation. The pH of HMJ changed significantly after LAB fermentation (Fig. 1B). HMJ had the highest pH, while HMJ fermented by H22B656 had much lower pH values due to greater lactic acid production. The results of TA were opposite to the trend of pH changes (Fig. 1C, *p* < 0.05). This acidification process is essential for inhibiting harmful microorganisms and improving product stability (Ibrahim et al., 2021). The levels of reducing sugar varied among the groups, with HNU329-fermented HMJ showing the highest amount, followed by HNU212-fermented HMJ, and H22B656-fermented HMJ showing the lowest (Fig. 1D). Moreover, the trend of soluble solids content of fermented HMJ was consistent with pH values (Fig. 1E). These findings indicated that *B. lactis* fermentation significantly altered the physicochemical properties of HMJ.

### 3.2 Organic acid profiles of HMJ and LAB fermented HMJ

**Fig. 2:**
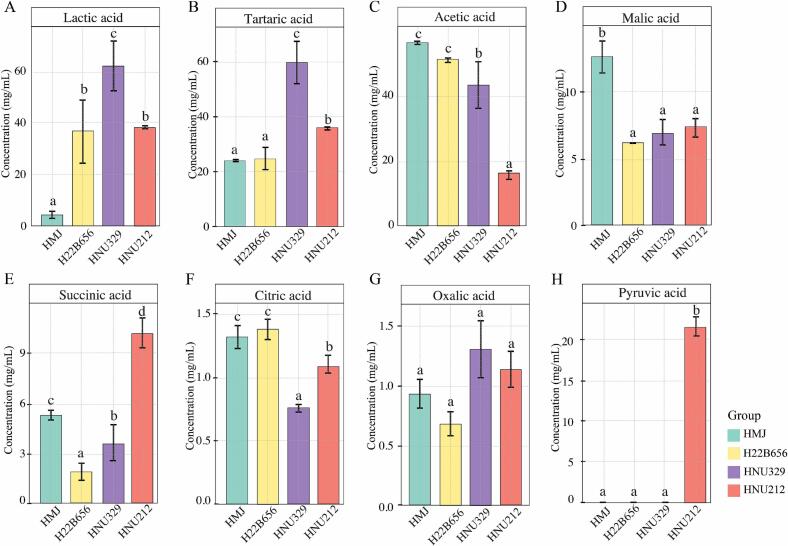
Organic acid profiles of HMJ and LAB fermented HMJ. Lactic acid (A); tartaric acid (B); acetic acid (C); malic acid (D); succinic acid (E); citric acid (F); oxalic acid (G); and pyruvic acid (H).

LAB fermentation produces various organic acids, including lactic acid, citric acid, acetic acid, succinic acid, and propionic acid, which help to prevent spoilage, improve food taste, and boost consumer acceptance and appeal (Bangar et al., 2022). The organic acid profiles of HMJ fermented by *B. lactis* are shown in Fig. 2. Lactic acid content was the most abundant organic compound in LAB-fermented HMJ, with its concentration significantly higher in HMJ fermented with HNU329, followed by HNU212 and H22B656 (Fig. 2A). The LAB strains are key factor in determining the lactic acid concentration produced during fermentation (Bangar et al., 2022). Tartaric acid, another predominant organic content in fermented HMJ, exhibited a high content after fermentation, and similar trend with lactic acid, which significant highest in HNU329-fermented HMJ and lowest in H22B656-fermented HMJ (Fig. 2B). As a byproduct of fermentation, tartaric acid, along with lactic acid, reflects microbial activity involved in sugar conversion. Additionally, previous research has shown that tartaric acid affected wine astringency by modifying protein-polyphenol interactions and altering protein structures through shifts in hydrogen and hydrophobic bonding (Zhao et al., 2023). Acetic acid exhibited an opposite trend compared to lactic and tartaric acid, showing a significant reduction in fermented HMJ. Acetic acid concentration was highest in H22B656-fermented HMJ and lowest in HNU212-fermented HMJ (Fig. 2C). Malic acid plays a key role in metabolic pathways, particularly in the tricarboxylic acid (TCA) cycle, and follows a similar trend with acetic acid, decreasing in fermented HMJ (Fig. 2D). HMJ fermented with HNU212 exhibited the greatest succinic acid content (Fig. 2E), which is an important key intermediate in the TCA cycle and the major product of microbial fermentation. HMJ fermented with H22B656 exhibited the highest concentration of citric acid, while HMJ fermented with HNU329 exhibited the lowest concentration (Fig. 2F). Citric acid is a key substance in the physiological oxidation of fats, proteins, and carbohydrates to carbon dioxide and water (Goldberg & Rokem, 2009). Similarly, HNU212- and HNU329-fermented HMJ exhibited elevated levels of oxalic acid (Fig. 2G), which is a promising natural compound with multiple benefits for improving the shelf life and quality of fruits and vegetables (Hasan et al., 2023). As an intermediate in sugar metabolism during fermentation, pyruvic acid plays a critical role in energy production and the overall metabolic process, and the elevated pyruvic acid content during fermentation may reflect the active degree of glycolysis (Schormann et al., 2019). However, pyruvic acid was only detected in the MFJ fermented with HNU212 (Fig. 2H). Meanwhile, pyruvic acid in H22B656-fermented juice, and HNU329-fermented juice may have been completely converted to lactic acid and other metabolites. These variations in organic acid concentrations highlight differences in microbial activity and fermentation efficiency across the samples. Additionally, fermentation promotes the biotransformation of organic acids, contributing to flavor customization and functional enhancement of fermented HMJs.

### 3.3 Bioactive substances and bioactivities of HMJ and fermented HMJ

**Fig. 3:**
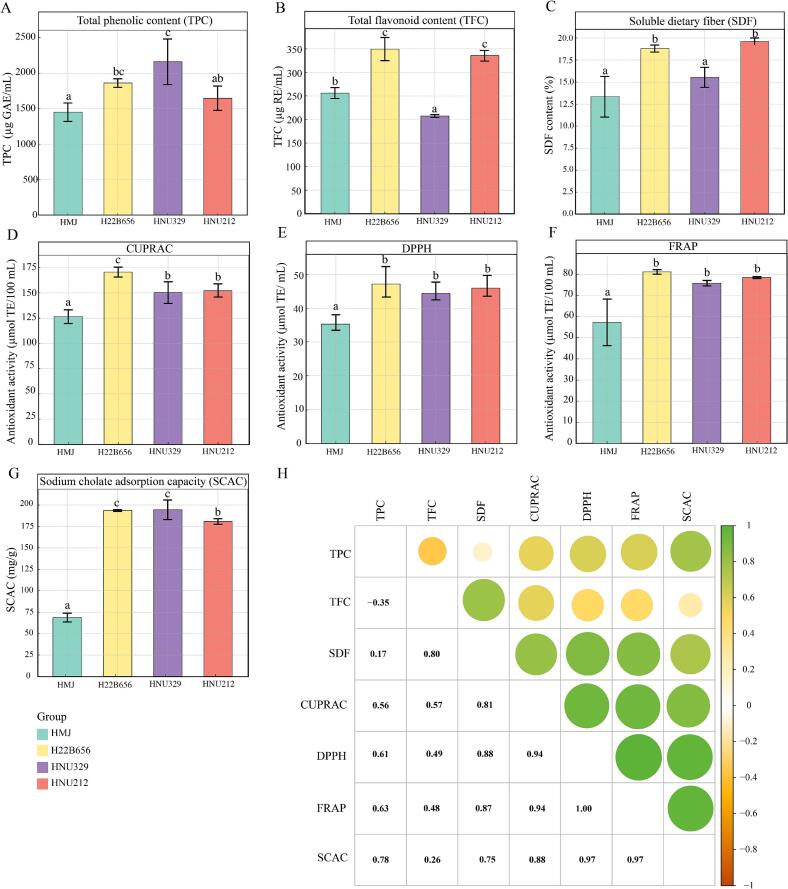
Change in bioactive substances and bioactive activities. Total phenolic content (TPC) (A); total flavonoid content (TFC) (B); soluble dietary fiber (SDF) (C); CUPRAC (D); DPPH (E); FRAP (F); sodium cholate adsorption capacity (SCAC) (G); and correlation of bioactive substances and bioactive activities (H) of HMJ and LAB fermented HMJ.

The bioactive substances and bioactivities of the *B. lactis*-fermented HMJ were analyzed. TPC was highest in HMJ fermented with HNU329 (2160.83 ± 321.23 μg GAE/mL), followed by H22B656 and HNU212, respectively Fig. 3A. Phenolic compounds are primary contributors to bioactivity in fermented products. The increase in TPC could be attributable to the hydrolysis of substantial polymeric phenolics into simple new phenolic compounds produced by LAB strains (Wang et al., 2021). As exhibited in Fig. 3B**,** H22B656-fermented HMJ (349.50 ± 24.56 μg RE/mL) had the highest TFC, followed by HNU212 (335.72 ± 10.78 μg RE/mL**)**, HMJ (256.11 ± 11.59 μg RE/mL), and HNU329 (207.44 ± 2.99 μg RE/mL). Flavonoids are bioactive substances that exhibit antioxidant, anticancer, antimicrobial, neuroprotective, and anti-inflammation activities (Al-Khayri et al., 2022). SDF is another plant essential component and has been widely recognized for its role in mitigating the risks of chronic diseases such as cardiovascular disease and colon cancer (Lin et al., 2024). All *B. lactis* -fermented HMJ exhibited an increase in SDF content, with HNU212, H22B656, and HNU329 fermented HMJ having SDF contents of 19.6 ± 0.4 %,18.8 ± 0.4 %, and 15.53 ± 1.13 %, respectively (Fig. 3C). Our findings support a prior report demonstrating that LAB fermentation enhances SDF content, as well as the physicochemical and functional properties in fermented okara (Wang, Xue, et al., 2024).

CUPRAC, DPPH, and FRAP assays are utilized to measure the antioxidant activity of the bioactive substances. As shown in Fig. 3D-F, antioxidant activities were increased in *B. lactis*-fermented HMJ, confirming that LAB strains possessed positive effects on the functional characteristic. All three assays exhibited a consistent trend, with the highest antioxidant activity observed in HMJ fermented with H22B656. The CUPRAC, DPPH, and FRAP assays showed a significantly higher antioxidant activity in H22B656-fermented HMJ (*p* < 0.05), while no significant differences were found between HNU329- and HNU212-fermented HMJ (Fig. 3D-F). The antioxidant activities are mainly attributed to their redox properties, which are crucial for adsorbing and neutralizing free radicals, quenching single and triple oxidants, or breaking down the peroxides. These antioxidant results align with the TFC (Fig. 3B), supporting previous studies that identified flavonoid as the primary bioactive substance with excellent antioxidant properties (Al-Khayri et al., 2022).

Furthermore, *B. lactis* fermentation enhanced SCAC of HMJ, and HMJ fermented with H22B656 and HNU329 exhibited significantly higher SCAC compared to HNU212-fermented HMJ (Fig. 3G). When sodium cholate levels decrease due to reduced reabsorption, the body automatically converts cholesterol into bile acids to compensate, thereby promoting cholesterol consumption and reducing overall cholesterol levels. This adaptive mechanism is essential to the liver-intestine-heart axis, which coordinates cholesterol homeostasis, lipid metabolism, inflammation, and metabolic diseases such as diabetes and obesity (Chiang et al., 2020). This finding suggests that *B. lactis* fermentation strengthens bile acid-binding properties in HMJ, supporting its potential as a functional food for cholesterol reduction.

Correlation analysis demonstrated a strong positive relationship between bioactive substances and bioactivities (Fig. 3H). The high correlation between TPC, TFC, and antioxidant activities indicates that phenolic compounds, including flavonoids, are the primary contributors to the antioxidant capacity of the samples (Zhang, Li, et al., 2023). These results are consistent with previous studies, which also observed similar correlations between TPC, TFC, and antioxidant activity in oats (Zhang, Li, et al., 2023), further supporting the significance of phenolic compounds in antioxidant defense mechanisms. Moreover, a strong correlation was observed between SDF and SCAC in *B. lactis*-fermented HMJ. This relationship implies that an increase in SDF content enhances SCAC, which is critical for lipid metabolism and cholesterol regulation. This finding indicates that the SDF in fermented HMJ had a potential hypolipidemic effect (Lin et al., 2024). Overall, *B. lactis* fermentation increases the levels of bioactive compounds, thereby enhancing bioactivities and potentially offering greater health benefits.

### 3.4 Metabolic profile of HMJ and LAB fermented HMJ

**Fig. 4:**
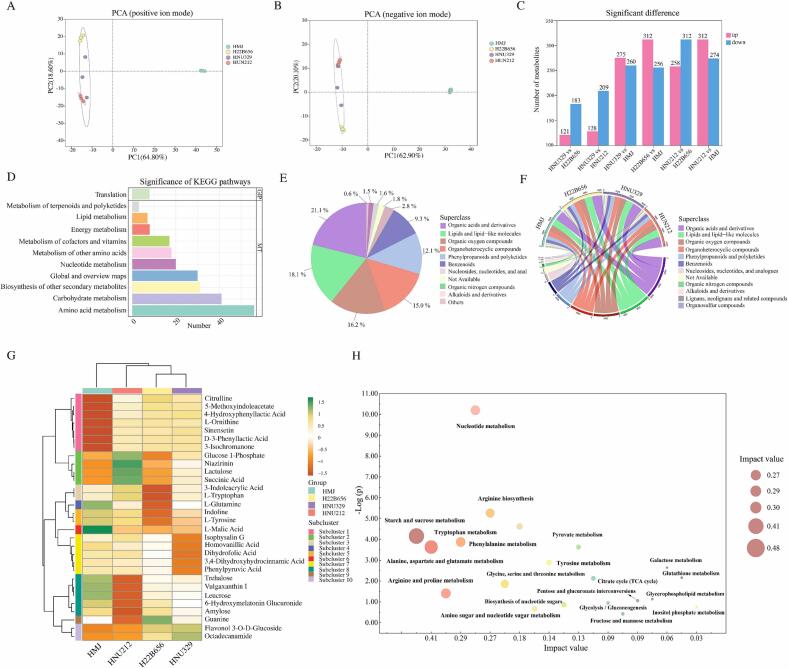
Non-target metabolomic analysis. Principal Component Analysis (PCA) plots of the metabolite profiles for positive ion mode (A); and negative ion mode (B); bar plot of significantly differential metabolites based on KEGG database (C); bar plot of the significance of KEGG pathways enriched in differential metabolites (D); pie chart of the overall distribution of differential metabolite superclass (E); chord diagrams of differential metabolites superclass across different groups based on the Human Metabolome Database (HMDB) (F); heatmap of the top 30 differential metabolites in different groups (G); and KEGG pathway topology analysis of top 20 metabolic pathways in differential metabolites (H).

Non-target metabolomics analysis was performed to examine the changes in the metabolic profile of unfermented and fermented HMJ. Similar clustering patterns were observed in the principal component analysis (PCA) plots in both ionic modes (Fig. 4A-B). HMJ appeared clearly separated from H22B656-, HNU329-, and HNU212-fermented HMJ, highlighting significant differences in metabolite compositions between the unfermented and fermented groups. Subsequently, differential metabolites were identified by calculating the VIP scores and *p*-values of all detected metabolites based on the OPLS-DA data. A total of 638 differential metabolites were identified, including 360 metabolites in positive ion mode and 278 metabolites in negative ion. Fig. 4C illustrated the changes in differential metabolites between groups. Among them, HMJ fermented by H22B656 and HNU212 revealed the highest number of up-regulated metabolites (*n* = 312) compared to unfermented HMJ. Bar plots illustrated the functional categories of differential metabolites based on the KEGG database (Fig. 4D). Amino acid metabolism was the most prominent pathway, followed by carbohydrate metabolism. The dominance of amino acid metabolism reflects the production of bioactive metabolites, such as citrulline and ornithine, which contribute to the nutritional and functional value of the samples (Aguayo et al., 2021; Sivashanmugam et al., 2017). Carbohydrate metabolism underscores the breakdown of sugars and the production of energy-yielding compounds, supporting microbial growth and acid production during fermentation (Fan & Pedersen, 2021).

The pie chart showed the distribution of different metabolites across different categories based on HMDB database (Fig. 4E), including organic acids and derivatives (21.1 %), lipids and lipid-like molecules (18.1 %), organic oxygen compounds (16.2 %), organoheterocyclic compounds (15.0 %), phenylpropanoids and polyketides (12.1 %), benzenoids (9.3 %), and other categories collectively (8.2 %). Organic acids and derivatives dominate the metabolite composition, consistent with research on LAB-fermented *Opuntia ficus-indica* fruit juice (Wang, He, et al., 2024). The organic acids are crucial for flavor development, pH regulation, and juice preservation. LAB plays a key role in promoting lipolysis, leading to the production of flavor precursors such as free fatty acids. Therefore, lipids and lipid-like molecules also contribute to the unique flavor of food products and positively influence the overall flavor of the finished product. (Zhang, Zhang, Guo, Bai, et al., 2023). As shown in Fig. 4F, the distribution of the differential metabolites in HMJ and three *B. lactis*-fermented HMJ groups varied across different classes based on the HMDB database. This result indicated that *B. lactis* reshaped the metabolite composition of HMJ through specific metabolic activities, and this difference in class distribution revealed the functional differentiation of the strains.

The heatmap highlights significant differences in 30 metabolites abundances of four groups (Fig. 4G). Subclusters in the heatmap further differentiate metabolites based on their distribution and relative abundances. *B. lactis* fermentation significantly elevated the levels of key differential metabolites, including citrulline, 5-methoxyindole acetate, 4-hydroxyphenyl acetic acid (4-HPAA), ornithine, sinensetin, and D-3-phenyllactic acid. Citrulline has various health benefits and is emerging as a natural alternative to pharmaceutical products for improving exercise performance and addressing several health conditions (Aguayo et al., 2021). 4-HPAA is a significant phenolic compound with potent antioxidant, antibacterial, and anti-inflammatory properties (Cheng et al., 2025). D-3-phenyllactic acid was increased after *B. lactis* fermentation, which is known for its antifungal activity and various beneficial pharmacological properties (Xu et al., 2021). Additionally, L-ornithine plays a key role in regulating several metabolic processes, and is linked to conditions such as cancer, hyperammonemia, and hyperornithinemia in humans (Sivashanmugam et al., 2017). Variations in metabolite profiles across groups highlight the influence of fermentation conditions and microbial strains in shaping the biochemical characteristics of the samples. Of note, we further subjected the differential metabolites to KEGG pathway topology analysis. The 20 metabolic pathways, as demonstrated in Fig. 4H, mainly contained amino acid metabolism and carbohydrate metabolism, in line with pathway enrichment analysis (Fig. 4D). Among them, the topological analysis showed that the metabolic pathways, such as starch and sucrose metabolism, TCA cycle, tryptophan metabolism, and galactose metabolism, were significantly enriched, which together affected the relevant biotransformation of HMJ metabolites before and after fermentation.

### 3.5 Overview of key metabolites and metabolic pathway changes

**Fig. 5:**
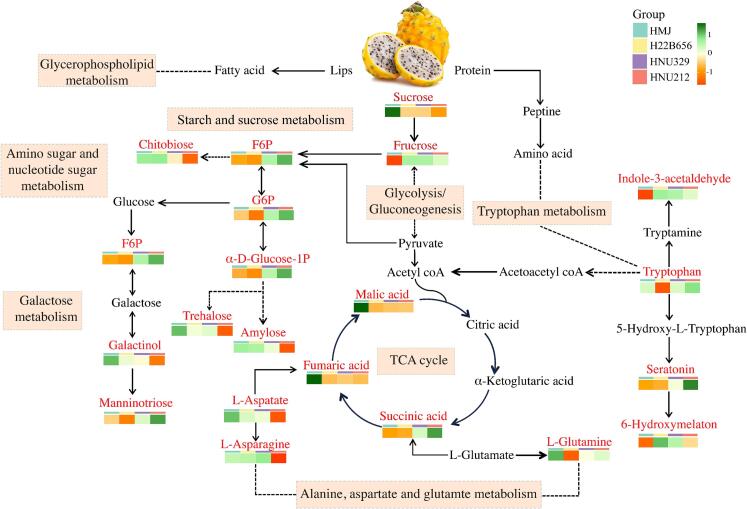
Overview of key metabolites and metabolic pathway changes in HMJ and LAB fermented HMJ. Substances marked in red are key differential metabolites and substances marked in black are intermediate metabolites of the metabolic pathway. (For interpretation of the references to color in this figure legend, the reader is referred to the web version of this article.)

It is well known that the metabolic processes of the three major nutrients (sugars, proteins, and lipids) and their interrelationships constitute a complex metabolic network. Together, these substances sustain the body's energy production and basic life activities. The transformation of metabolites during the fermentation of plant-based products is highly significant for consumers promoting a balanced diet and healthy living. This study analyzed the differential metabolites biotransformation in enriched metabolic pathways (Fig. 5). Key pathways affected include starch and sucrose metabolism, amino sugar and nucleotide metabolism, galactose metabolism, glycolysis, TCA cycle, and tryptophan metabolism. Glycerophospholipid metabolism and glycolipid metabolism are closely related through shared precursor substances (e.g., fatty acids, glycosyl donors), intersecting metabolic nodes (e.g., phosphatidic acid), and common regulatory mechanisms (e.g., insulin signaling). These associations not only maintain cellular metabolic homeostasis in normal physiological states but also play important roles in disease states such as diabetes and obesity (Chiang et al., 2020). In starch and sucrose metabolism, *B.lactis* fermentation resulted in the breakdown of sucrose in HMJ into glucose, fructose, and other metabolites through enzymatic reactions. The breakdown of sucrose and amylose during fermentation contributes to the accumulation of simpler sugars, supporting microbial metabolism and energy production. Galactose enters central metabolic pathways through the Leloir pathway, and then is converted into glucose-6-phosphatase (G6P). From there, G6P can enter the pentose phosphate pathway, allowing galactose to be integrated into energy production and cellular biosynthesis. Additionally, galactinol and manninotriose are more specific to plant metabolism and carbohydrate storage. Meanwhile, the increasing number of rare congenital disorders associated with galactose, along with its emerging therapeutic applications, is drawing growing attention to galactose metabolism (Conte et al., 2021). Our findings confirmed fermentation enhances the glycolytic pathway evidenced by increased levels of G6P and fructose-6-phosphate (F6P) in fermented groups.

After glycolysis, the pyruvate molecules are transported into the mitochondria. There, each pyruvate is decarboxylated by the pyruvate dehydrogenase complex (PDC) into acetyl-CoA, and then acetyl-CoA enters the TCA cycle. The TCA cycle plays a crucial role in the production of organic acids and has been extensively used in many fields (Zhou et al., 2023). Changes in the content of TCA cycle intermediates, including malic acid, fumaric acid, and succinic acid, were consistent with the results of the organic acid assay, malic acid content in HMJ significantly reduced after *B. lactis* fermentation, suggesting its conversion to lactic acid through the malolactic enzyme. Additionally, LAB may facilitate the transformation of fumaric acid to succinic acid or other metabolites, leading to a reduction in fumaric acid content in HMJ fermented by HNU329 (Ma et al., 2025).

Amino acid metabolites can enter the TCA cycle and contribute to the oxidative breakdown of fatty acids. Similarly, intermediate products of sugar metabolism (e.g., oxaloacetic acid) serve as carbon scaffolding for amino acid synthesis. As shown in Fig. 5, tryptophan metabolism is explored as a starting point to examine changes in amino acid metabolites in HMJ before and after fermentation. Notably, the levels of tryptophan-derived metabolites, including serotonin and 6-hydroxy melatonin, were elevated in fermented juice. This highlights the potential role of lactic acid bacteria in modulating bioactive compounds with significant health benefits, such as improved sleep regulation and mood enhancement of tryptophan-derived metabolites (Eugster et al., 2022). Tryptophan metabolism produces a variety of bioactive compounds know to influence inflammation, metabolism, immune responses, and neurological function (Xue et al., 2023). Among these, indole-3-acetaldehyde (IAAD) levels were significantly up-regulated after *B. lactis* fermentation, reflecting the positive biotransformation of indole in tryptophan metabolism. IAAD has been reported to play a regulatory role in immune function by activating aryl hydrocarbon receptors (AhR), reinforcing its potential health-promoting effects (Dai et al., 2024). Collectively, these results indicate that *B. lactis*-mediated fermentation not only modifies critical metabolic pathways in HMJ but also potentially improves its bioactivity by enriching health-relevant tryptophan metabolites.

### 3.6 Sugar compounds profile of HMJ and LAB fermented HMJ

**Fig. 6:**
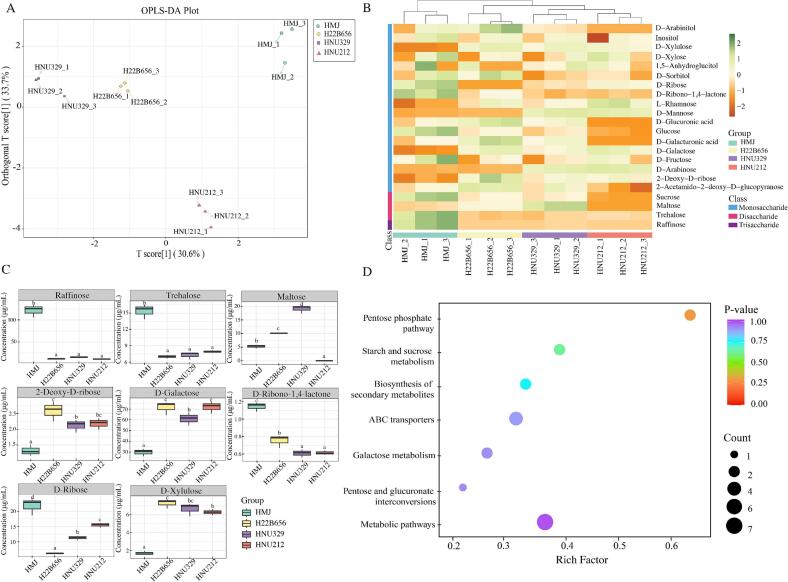
Analysis of targeted sugar compounds Orthogonal Partial Least Squares Discriminant Analysis (OPLS-DA) score plots of sugar composition (A); heatmap cluster analysis of all sugar compounds (B); changes in the content of eight differential sugar compounds (C); and KEGG pathway enrichment analysis based on the differential sugar compounds results (D).

Dietary fiber can simultaneously improve abnormal carbohydrate metabolism (e.g., diabetes mellitus) and lipid metabolism disorders (e.g., hypercholesterolemia) by delaying sugar absorption and adsorbing cholesterol (Lin et al., 2024). *Hylocereus megalanthus* is naturally rich in dietary fiber, and *B. lactis* fermentation significantly increased SDF content and enhanced SCAC. Non-targeted metabolomics analysis also revealed that differential metabolites in *B. lactis* -fermented HMJ were primarily enriched in the carbohydrate metabolism pathway. Consequently, this study focuses on sugar metabolism within the carbohydrate metabolism pathway to explore the biotransformation of glycoconjugates in HMJ before and after fermentation. The sugar composition of a fruit plays a crucial role in defining flavor, taste, and overall consumer appeal (Zhang et al., 2022). A total of 22 sugar compounds, including monosaccharides, disaccharides, and trisaccharides, were detected in both fermented and unfermented HMJ using GC–MS analysis. As shown in the OPLS-DA score plots (Fig. 6A), the different HMJ groups were distinctly separated along the t1 axis, indicating the significant differences in their sugar metabolite profiles. Fig. 6B demonstrated the clustering of metabolites and samples, further emphasizing the metabolic differences among the fermentation groups. Moreover, eight differential metabolites were identified based on the VIP > 1 in the OPLS-DA model, with a significance threshold of *p* < 0.05. The variations in their concentrations are illustrated in Fig. 6C.

For monosaccharides, the contents of D-galactose and D-Xylulose increased significantly after fermentation. Notably, D-galactose plays a role in the synthesis of molecules essential for neural health, including components of the brain's extracellular matrix, which help support neural function and communication. Through a series of enzymatic reactions, D-galactose can be converted to G6P, entering either the glycolytic or pentose phosphate pathway (Jagtap et al., 2019). Simultaneously, microbial activity converts D-xylose into D-xylulose through xylose isomerase, an important precursor of arabinitol that plays a role in metabolic regulation. Interestingly, alginate and maltose, both disaccharides, showed opposite trends in concentration after fermentation, probably due to the metabolism of lactobacilli enzymatic activity facilitating their interconversion. In addition, fermentation led to a reduction in the functional oligosaccharide raffinose (*p* < 0.05), which plays a key role in promoting *Bifidobacteria* colonization (Wang, Li, et al., 2022). During fermentation, certain microorganisms secrete α-galactosidase, hydrolyzing raffinose into monosaccharides (e.g., glucose and galactose), which subsequently undergo glycolysis to produce energy and metabolites (e.g., lactic acid and ethanol). These metabolic transformations influence the quality and flavor of the fermented juice (Wang, Li, et al., 2022). Notably, all three *B. lactis*-fermented HMJ showed similar glycoconjugate modification patterns.

KEGG pathway enrichment was performed based on the differential metabolite, where the rich factor represents the ratio of the number of differential metabolites within each pathway (Fig. 6D). The results indicated significant enrichment of differential metabolites in the pentose phosphate pathway (PPP). The PPP is closely related to the TCA cycle and the gluconeogenesis pathway, facilitating metabolic redistribution through the transfer of intermediates. Furthermore, PPP metabolites contribute to the production of acetylphosphate and glyceraldehyde 3-phosphate, thus affecting both the yield and composition of fermentation products (Abdel-Rahman et al., 2013). During LAB fermentation, the *Bifidobacterium* mainly utilizes the bifid shunt as its core metabolic pathway, and the final products are acetate and lactate. In addition, metabolic pathways such as “starch and sucrose metabolism”, “galactose metabolism”, and “pentose and glucuronate interconversions” are integral to *B. lactis* fermentation, contributing to carbohydrate metabolism and regulation of the fermentation process.

### 3.7 Sensory profile analysis of HMJ and LAB fermented HMJ

**Fig. 7:**
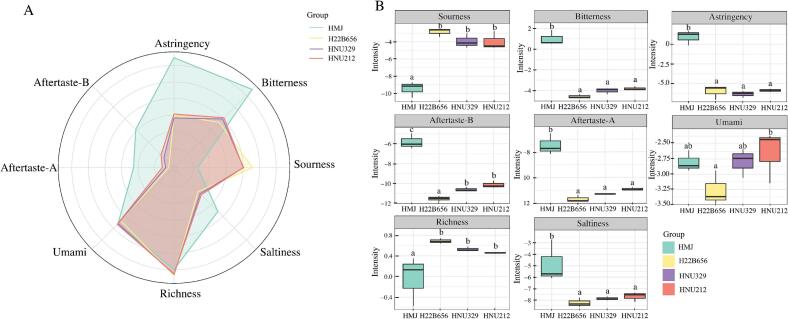
E-tongue radar chart (A) and variation of flavor profile (B) of HMJ and LAB fermented HMJ.

By detecting the basic flavor components (e.g., sour, sweet, bitter, salty, fresh, etc.), the e-tongue offers a rapid and objective method for assessing the sensory qualities of fermented juices. In this study, the E-tongue was used to compare the astringency, bitterness, sourness, saltiness, richness, umami, aftertaste-A, and aftertaste-B levels of HMJ before and after fermentation (Fig. 7A). Changes in each flavor are shown in Fig. 7B. Among them, sourness level was lower in unfermented HMJ, reflecting its unfermented nature. These changes were related to the pH value, which decreased after fermentation (Fig. 1A). Consistent with the E-tongue analysis, sensory evaluation results showed a significant increasing of sourness after fermentation. In contrast, fermented HMJ exhibited enhanced color, odor, and overall acceptability (Fig. S1). These findings further support that fermentation promotes sugar utilization and organic acids production, collectively improving the overall flavor profile. A similar trend was observed for richness level, which increased after fermentation. This finding is consistent with previous research, such as fermented goji juice (Duan et al., 2023). As expected, fermentation markedly reduced bitterness, astringency, aftertaste-A, aftertaste-B, and saltiness levels of HMJ. Notably, the three *B. lactis*-fermented HMJs expressed consistent fermentation properties, which were associated with changes in the compounds of interest during fermentation. For instance, bitterness and astringency levels were strongly correlated with soluble sugars, lactic acid, and acetic acid in fermented radishes (Deba-Rementeria et al., 2023). Additionally, the decrease in tyrosine, an amino acid associated with bitterness, also contributed to these sensory characteristics (Liu et al., 2024). HMJ, as the unfermented baseline, demonstrates the transformative impact of fermentation on the sensory attributes of the fruit. These differences suggest that specific bacterial strains used in fermentation uniquely influence flavor development and the metabolites that affect the flavors (Duan et al., 2023). Therefore, lactic acid fermentation induces complex metabolic changes that enhance flavor complexity and sensory appeal.

## 4 Conclusion

Overall, the fermentation characteristics, biological activity, metabolic profile, and sensory properties of HMJ were significantly enhanced by *B. lactis* fermentation. After fermentation, the viable count (8.99–9.16 log CFU/mL) confirmed HMJ as a suitable substrate for probiotic fermentation. Fermentation enhanced acidity, reduced sugar content, and increased levels of TPC, TFC, and SDF, leading to elevated antioxidant activity and SCAC. These findings support the potential of fermented HMJ as a cholesterol-lowering product. Metabolomic analysis revealed substantial changes in the amino acid and carbohydrate metabolism pathways, including increased levels of functional metabolites such as 5-methoxyindole acetate, 4-hydroxyphenylacetic acid, ornithine, sinensetin, and D-3-phenyllactic acid. These compounds are associated with antioxidant, anti-inflammatory, and gut-regulatory functions. The improved sensory profile, confirmed by *E*-tongue and sensory evaluation, further supports its consumer acceptability. In conclusion, *B. lactis*-fermented HMJ represents a promising candidate for development as a functional beverage which potential benefits for gut health and metabolic wellness. These findings provide the functional food industry with significant information, particularly in the formulation of plant-based probiotic beverages targeting health-conscious consumers.

## CRediT authorship contribution statement

**Patthanan Sakda:** Writing – original draft, Software, Data curation. **Huixian Wang:** Writing – review & editing, Methodology, Conceptualization. **Juanni Li:** Methodology, Investigation, Data curation. **Jiangyue Zheng:** Software, Investigation. **Yuke He:** Writing – review & editing. **Wanwipa Vongsangnak:** Resources. **Ruimin Wang:** Writing – review & editing, Visualization, Supervision, Project administration.

## Declaration of competing interest

The authors declare that they have no competing financial interests or personal relationships that could have influenced the work report in this paper.

## Data Availability

Data will be made available on request.

## Appendix A Supplementary data

Supplementary material Image 1
