# Supplementary figures and images for: Enhancing functional properties and analysis of sugar and metabolite composition of Hylocereus megalanthus juice through Bifidobacterium fermentation
Source: Food Chem X. 2025 Aug 21;30:102945. doi: 10.1016/j.fochx.2025.102945 (PMC12409454; doi:10.1016/j.fochx.2025.102945)

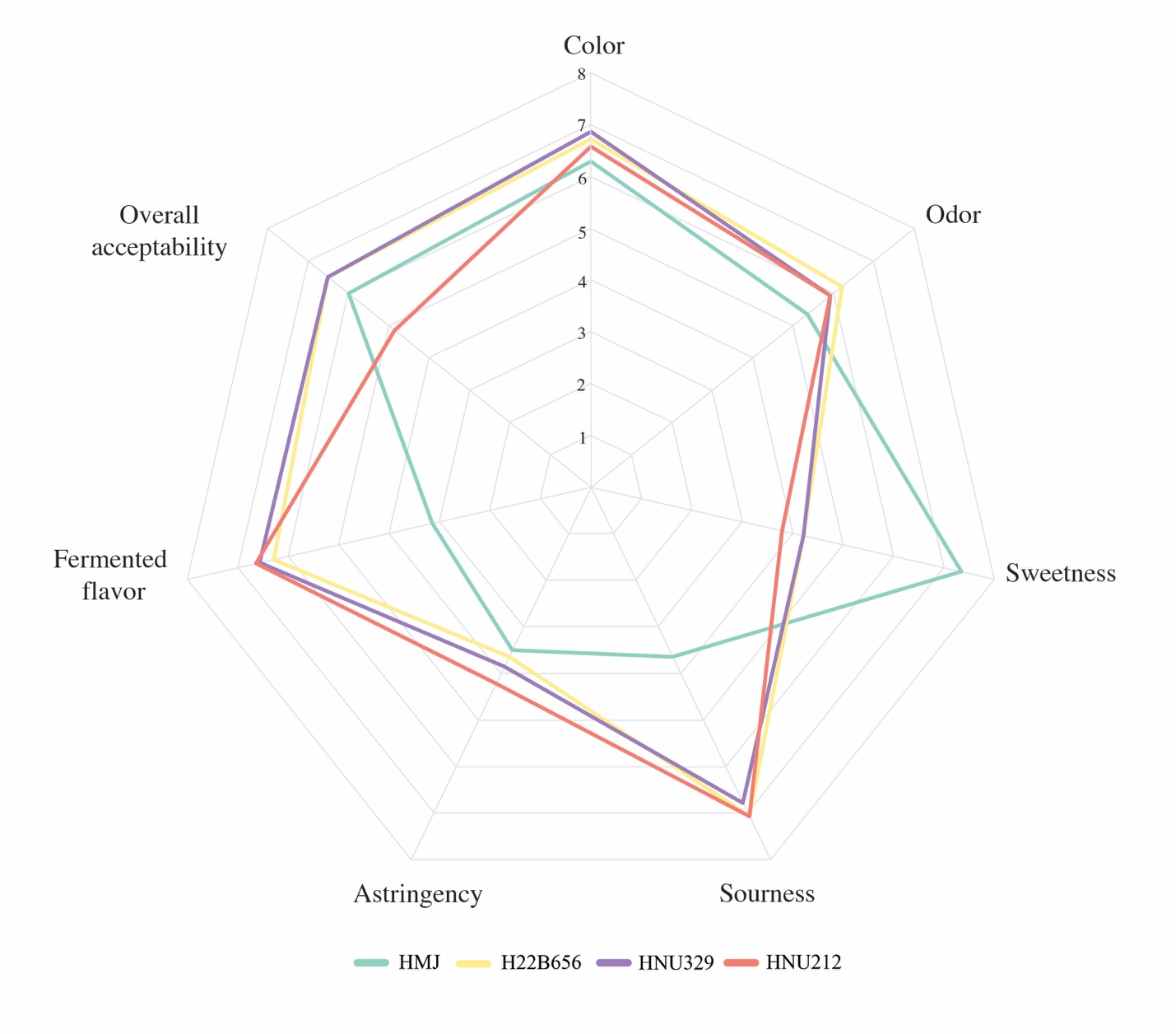


Fig. S1. Sensory evaluation radar chart of fermented and unfermented HMJ.

Supplement: Supplementary file 1 — Supplementary material [file mmc1.docx]
